# Genetic Variants in *NOS2* and *CCL2* Modulate Risk of Post‐COVID‐19 Hyperglycemia via Immune‐Metabolic Interactions

**DOI:** 10.1155/bmri/7994653

**Published:** 2026-07-30

**Authors:** Ganyalak Chaimaha, Nipaporn Teerawattanapong, Kaweeraphat Chaithaisong, Tassanee Narkdontri, Suavaluk Songlilitchuwong, Saranya Innang, Sarocha Suthon, Alessia Micieli, Watip Tangjittipokin

**Affiliations:** ^1^ Graduate Program in Immunology, Department of Immunology, Faculty of Medicine Siriraj Hospital, Mahidol University, Bangkok, Thailand, mahidol.ac.th; ^2^ Siriraj Center of Research Excellence for Diabetes and Obesity, Faculty of Medicine Siriraj Hospital, Mahidol University, Bangkok, Thailand, mahidol.ac.th; ^3^ Research Division, Faculty of Medicine Siriraj Hospital, Mahidol University, Bangkok, Thailand, mahidol.ac.th; ^4^ Medical Program, Faculty of Medicine Siriraj Hospital, Mahidol University, Bangkok, Thailand, mahidol.ac.th; ^5^ Vita-Salute San Raffaele University, Milan, Italy, unisr.it; ^6^ Department of Immunology, Faculty of Medicine Siriraj Hospital, Mahidol University, Bangkok, Thailand, mahidol.ac.th

**Keywords:** COVID-19, genetic polymorphism, hyperglycemia, postviral diabetes

## Abstract

Hyperglycemia has increasingly been recognized among individuals recovering from coronavirus disease 2019 (COVID‐19); the host factors contributing to heterogeneous metabolic outcomes remain poorly understood. This cohort study investigated the association between innate immune genetic polymorphisms and hyperglycemia in post‐COVID‐19 patients. A total of 471 adults with previous mild‐to‐moderate COVID‐19 were enrolled through the post‐COVID follow‐up program at Siriraj Hospital, Thailand, and classified as normoglycemic (HbA1c < 5.7%, *n* = 252) or hyperglycemic (HbA1c ≥ 5.7%, *n* = 219). Clinical characteristics, metabolic parameters, inflammatory biomarkers, and immunological measurements were collected during follow‐up. Genotyping was performed using the Axiom Human Genotyping SARS‐CoV‐2 Research Array, focusing on candidate polymorphisms in innate immune‐related genes. Associations between genetic variants and hyperglycemia were evaluated using multivariable logistic regression adjusted for age, body mass index, and underlying comorbidities. Participants with hyperglycemia were significantly older and had higher body mass index, greater prevalence of cardiometabolic comorbidities, elevated C‐reactive protein levels, and higher LDL cholesterol compared with normoglycemic individuals. Significant associations were identified in *NOS2* and *CCL2*. The *NOS2* rs4795067 variant was associated with increased odds of hyperglycemia (adjusted OR = 1.770, 95% CI: 1.137–2.756, *p* = 0.011), whereas rs35051118 showed a protective effect (adjusted OR = 0.613, 95% CI: 0.386–0.975, *p* = 0.039). In *CCL2*, rs28730833 was strongly associated with hyperglycemia (adjusted OR = 2.977, 95% CI: 1.349–6.572, *p* = 0.007). These findings suggest that innate immune genetic variation may contribute to hyperglycemia in post‐COVID‐19 patients, although independent validation is required.

## 1. Introduction

The long‐term consequences of coronavirus disease 2019 (COVID‐19) have emerged as a major public health challenge, extending beyond acute respiratory illness to include persistent multisystem complications [1]. Among these, metabolic abnormalities, particularly new‐onset hyperglycemia and worsening glycemic control, have received increasing attention due to their potential impact on long‐term morbidity and cardiovascular risk [2]. Clinical studies have reported that disturbances in glucose metabolism may persist for weeks to months after SARS‐CoV‐2 infection, even in individuals without previously diagnosed diabetes, suggesting that COVID‐19 may interact with host metabolic vulnerability in complex ways [3–6].

Hyperglycemia is a multifactorial metabolic disorder resulting from interactions between environmental exposures, host physiology, and genetic susceptibility [7]. Chronic low‐grade inflammation is well recognized as a central contributor to insulin resistance and *β*‐cell hyperfunction, with proinflammatory cytokines and chemokines capable of disrupting glucose homeostasis through impaired insulin signaling, oxidative stress, and altered immune‐metabolic communication [8–10]. Viral infections may further amplify these mechanisms by triggering sustained inflammatory responses, potentially unmasking latent metabolic hyperfunction in susceptible individuals [11].

Host genetic variation may partially explain why metabolic outcomes after infection differ substantially between individuals. Single‐nucleotide polymorphisms (SNPs), the most common form of genetic variation, have been associated with susceptibility to diabetes, inflammatory disorders, and differential immune responses to infection [12, 13]. In the context of COVID‐19, genetic determinants of immune regulation have been increasingly investigated for their role in disease severity and postinfectious complications; however, their contribution to post‐COVID metabolic hyperregulation remains insufficiently characterized.

Among candidate genes, *NOS2* and *CCL2* are biologically plausible mediators linking inflammation and metabolic hyperfunction [14, 15]. *NOS2* encodes inducible nitric oxide synthase (iNOS), a key enzyme responsible for nitric oxide production during inflammatory responses. Excessive iNOS activation has been associated with oxidative stress, impaired insulin signaling, *β*‐cell injury, and metabolic inflammation [16, 17]. Meanwhile, *CCL2* encodes monocyte chemoattractant protein‐1 (MCP‐1), a chemokine that promotes monocyte recruitment and macrophage infiltration into metabolically active tissues such as adipose tissue, thereby contributing to chronic inflammation and insulin resistance [18, 19]. Genetic variation in these pathways may therefore influence individual susceptibility to hyperglycemia following inflammatory stress.

Specific variants, in *NOS2* and *CCL2*, are of particular interest because of their potential functional relevance in regulating inflammatory responses [20], although their role in post‐COVID metabolic outcomes has not been previously established [21]. Identifying such variants may improve our understanding of host susceptibility to postinfectious metabolic complications and help refine risk stratification strategies.

Therefore, this study is aimed at investigating the association between polymorphisms in innate immune response genes, with a particular focus on *NOS2* and *CCL2*, and hyperglycemia in post‐COVID‐19 patients. By examining the interface between host genetics, immune activation, and metabolic outcomes, this study seeks to provide new insight into the biological mechanisms underlying post‐COVID hyperglycemia.

## 2. Subjects and Methods

### 2.1. Study Design and Participants

This prospective cohort study investigated the association between innate immune genetic polymorphisms and hyperglycemia among individuals recovering from COVID‐19 infection between August and November 2022 (COA No. Si 225/2022). Participants were recruited from the post‐COVID follow‐up program at Siriraj Hospital, Mahidol University, Bangkok, Thailand. Eligible participants were adults with laboratory‐confirmed SARS‐CoV‐2 infection diagnosed by reverse transcription polymerase chain reaction (RT‐PCR) who had recovered from mild‐to‐moderate COVID‐19. Patients with severe or critical illness requiring intensive care were excluded to minimize confounding from critical illness–associated metabolic disturbances, including stress hyperglycemia and treatment‐related metabolic effects.

**Figure 1:**
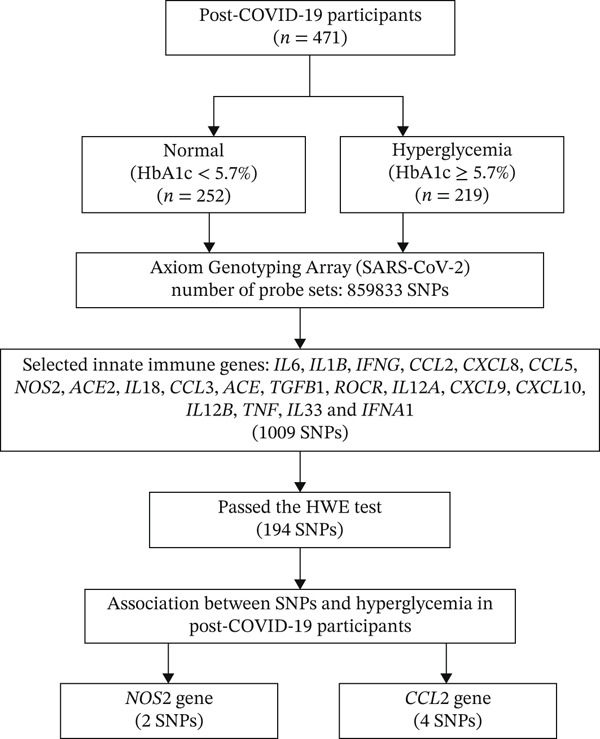
Overview of data analysis.

A total of 471 participants with complete clinical and genetic data were included in the final analysis (Figure 1). Glycemic status was classified according to glycated hemoglobin (HbA1c) measured during follow‐up, with participants categorized as normoglycemic (HbA1c < 5.7%) or hyperglycemic (HbA1c ≥ 5.7%) based on American Diabetes Association criteria. This definition was selected to capture the broader spectrum of impaired glucose regulation, including both prediabetic and diabetic phenotypes. Because the primary objective was to identify host genetic factors associated with differential metabolic outcomes within post‐COVID patients, a healthy noninfected control group was not included.

### 2.2. Clinical and Laboratory Data Collection

Clinical and demographic data were obtained from electronic medical records and standardized follow‐up assessments, including age, sex, body weight, height, body mass index (BMI), and underlying comorbidities. Laboratory evaluations included metabolic, inflammatory, and immunological biomarkers relevant to post‐COVID‐19 recovery. Metabolic markers comprised HbA1c, serum creatinine, and direct low‐density lipoprotein (LDL) cholesterol. HbA1c was used to assess long‐term glycemic status, whereas serum creatinine and LDL cholesterol were measured to evaluate renal function and lipid metabolism, respectively. Systemic inflammation was assessed using C‐reactive protein (CRP) and interleukin‐6 (IL‐6), both of which have been associated with immune activation and metabolic dysfunction. Humoral immune responses were evaluated by measuring neutralizing antibody activity and anti–receptor‐binding domain immunoglobulin G (anti‐RBD IgG), reflecting functional neutralizing capacity and SARS‐CoV‐2‐specific antibody responses.

### 2.3. DNA Extraction and Genotyping

Peripheral venous blood samples were obtained from all 471 participants and immediately processed by centrifugation to separate plasma, buffy coat, and red blood cells. The samples were stored at −80°C until further analysis. Genomic DNA was isolated from the buffy coat using the FlexiGene DNA Kit (Qiagen, Valencia, California, United States [Catalog No. 51206]) according to the manufacturer′s instructions. The purity and concentration of the extracted DNA were assessed using a NanoDrop 2000c spectrophotometer (Thermo Scientific, United States), ensuring suitability for downstream genotyping. DNA quality and concentration were assessed before analysis. Genotyping was performed using the Axiom Human Genotyping SARS‐CoV‐2 Research Array (Thermo Fisher Scientific, United States [Catalog No. 952402]), a high‐density array containing allele‐specific oligonucleotide probes targeting genome‐wide and immune‐related genetic variants associated with SARS‐CoV‐2 infection and host immune responses (ArrayExpress Accession: A‐MTAB‐716). Genotyping was performed according to the manufacturer′s validated protocol, including whole‐genome amplification, DNA fragmentation, hybridization, fluorescent staining, and automated imaging using the GeneTitan Multi‐Channel Instrument. Genotype calling was performed using the Axiom Analysis Suite software following the manufacturer′s recommended workflow.

### 2.4. Candidate SNP Selection and Quality Control

A targeted candidate gene approach was used to evaluate polymorphisms in innate immune response genes, with particular focus on *NOS2* and *CCL2* due to their biological relevance to inflammation and metabolic regulation. Quality control procedures included exclusion of samples with poor DNA quality, removal of low‐confidence variants, genotype quality filtering, and Hardy–Weinberg equilibrium testing to minimize technical artifacts. Only SNPs passing all quality control criteria were included in downstream analyses.

### 2.5. Statistical Analysis

Statistical analyses were performed using IBM SPSS Statistics Version 29.0 (SPSS Inc., Chicago, Illinois, United States). Continuous variables were assessed for normality before analysis to determine the most appropriate statistical approach. Normally distributed variables are presented as mean ± standard deviation (SD), whereas nonnormally distributed variables are reported as median with interquartile range (IQR). Categorical variables are expressed as frequencies and percentages.

Comparisons between normoglycemic and hyperglycemic groups were performed using independent samples *t*‐tests for normally distributed continuous variables and Mann–Whitney *U* tests for nonnormally distributed data. Categorical variables were analyzed using Pearson′s chi‐square test or Fisher′s exact test, depending on cell distribution.

Associations between candidate SNPs and hyperglycemia were evaluated using binary logistic regression analysis. Initial crude models were constructed to estimate unadjusted associations between genotype or allele frequencies and glycemic status. Multivariable logistic regression models were subsequently adjusted for clinically relevant confounding factors, including age, BMI, and underlying comorbid diseases, given their established influence on metabolic hyperfunction and glucose regulation. Odds ratios (ORs) with corresponding 95% confidence intervals (CIs) were calculated to quantify the strength of association. A two‐sided *p* value < 0.05 was considered statistically significant.

Because multiple testing correction was not applied in this exploratory candidate gene analysis, statistically significant associations should be interpreted with appropriate caution. These findings are therefore considered hypothesis‐generating rather than definitive and require confirmation in independent external cohorts.

## 3. Results

### 3.1. Clinical Characteristics of the Study Population

A total of 471 post‐COVID‐19 participants were included in the analysis, comprising 252 normoglycemic individuals (HbA1c < 5.7%) and 219 participants classified as hyperglycemic (HbA1c ≥ 5.7%). Comparisons of baseline clinical characteristics demonstrated significant metabolic differences between the two groups (Table 1). Participants in the hyperglycemia group were significantly older than normoglycemic individuals (median 54.66 vs. 39.81 years, *p* < 0.001) and had higher body weight (68.0 vs. 62.0 kg, *p* < 0.001) and BMI (26.33 vs. 23.53 kg/m^2^, *p* < 0.001) (Table 1). Although height differed slightly between groups (*p* = 0.029), no significant difference was observed in sex distribution (*p* = 0.804). Assessment of laboratory parameters revealed persistent metabolic and inflammatory differences between groups (Table 1). Participants with hyperglycemia exhibited significantly higher HbA1c levels at both 1 month (*p* < 0.001) and 3 months (*p* < 0.001), consistent with sustained glycemic hyperregulation. Inflammatory burden also appeared greater in the hyperglycemia group, as reflected by higher CRP levels at 3 months (1.71 vs. 1.36 mg/dL, *p* = 0.008). Direct LDL cholesterol levels were similarly elevated in hyperglycemic participants (129 vs. 117 mg/dL, *p* = 0.008). No statistically significant differences were observed in neutralizing antibody responses, anti‐RBD IgG levels, IL‐6 concentrations, or serum creatinine between groups. These findings indicate that hyperglycemia in post‐COVID participants was associated with an adverse metabolic profile and evidence of persistent low‐grade inflammation.

**Table 1:** Characteristics of the study population.

Clinical characteristics	Control (HbA1c < 5.7) (*n* = 252)	Case (HbA1c ≥ 5.7) (*n* = 219)	*p*value
Age (years)	39.81 (28.89, 51.52) (*n* = 252)	54.66 (43.01, 62.08) (*n* = 219)	< 0.001
Weight (cm)	62.0 (53.3, 72.0) (*n* = 252)	68.0 (58.0, 78.0) (*n* = 219)	< 0.001
Height (cm)	160 (156, 168) (*n* = 252)	160 (153, 167) (*n* = 219)	0.029
BMI (kg/m^2^)	23.53 (20.55, 27.62) (*n* = 252)	26.33 (23.84, 29.14) (*n* = 219)	< 0.001
Male, *n* (%)	95 (38.1%)	81 (37%)	0.804
Nab follow‐up 1 month (% inhibition)	97.68 (97.03, 98.15) (*n* = 30)	97.82 (97.33, 98.26) (*n* = 14)	0.288
Nab follow‐up 3 months (% inhibition)	96.82 (95.14, 97.59) (*n* = 251)	96.92 (95.59, 97.60) (*n* = 218)	0.249
CRP follow‐up 1 month (mg/dL)	1.16 (0.60, 3.89) (*n* = 30)	1.57 (0.60, 3.21) (*n* = 14)	0.625
CRP follow‐up 3 months (mg/dL)	1.36 (0.64, 2.80) (*n* = 252)	1.71 (0.83, 3.45) (*n* = 218)	0.008
HbA1c follow‐up 1 month (%)	5.40 (5.28, 5.60) (*n* = 30)	6.80 (6.30, 8.25) (*n* = 14)	< 0.001
HbA1c follow‐up 3 months (%)	5.4 (5.2, 5.5) (*n* = 252)	6.1 (5.8, 6.6) (*n* = 219)	< 0.001
Cr follow‐up 1 month (mg/dL)	0.77 ± 0.19 (*n* = 30)	0.79 ± 0.21 (*n* = 14)	0.793
Cr follow‐up 3 months (mg/dL)	0.74 (0.62, 0.93) (*n* = 252)	0.75 (0.61, 0.98) (*n* = 218)	0.368
Direct LDL cholesterol follow‐up 1 month (mg/dL)	123 (100, 164.50) (*n* = 30)	120.50 (97.75, 152.25) (*n* = 14)	0.588
Direct LDL cholesterol follow‐up 3 months (mg/dL)	117 (96, 143) (*n* = 252)	129 (102, 156) (*n* = 218)	0.008
Anti‐RBD IgG level follow‐up 1 month (AU/mL)	8060.3 (2808.6, 17,766.9) (*n* = 250)	8577.7 (4160.9, 17,137.3) (*n* = 218)	0.202
Anti‐RBD IgG level follow‐up 3 months (AU/mL)	7110.9 (2796.5, 14,585.0) (*n* = 252)	8168.5 (4160.9, 17,137.3) (*n* = 218)	0.063
IL‐6 follow‐up 1 month (pg/mL)	1.50 (1.50, 1.50) (*n* = 250)	1.5 (1.50, 1.69) (*n* = 218)	0.088
IL‐6 follow‐up 3 months (pg/mL)	1.50 (1.50, 2.15) (*n* = 30)	1.54 (1.50, 2.14) (*n* = 13)	0.666

*Note:* Data showed mean ± SD or median (Q1, Q3) according to data distribution. *p* value < 0.05 was considered statistically significant.

Abbreviations: AU/mL, arbitrary concentration units; BMI, body mass index; Cr, creatinine; CRP, C‐reactive protein; IL‐6, interleukin‐6; Nab, neutralizing antibody.

### 3.2. Comorbidity Profiles

Because underlying metabolic health may substantially influence glycemic outcomes, comorbidity profiles were compared between groups (Table 2). Participants in the hyperglycemia group demonstrated a significantly higher prevalence of established cardiometabolic comorbidities.

**Table 2:** Comorbidities of the study population.

Comorbidities	Control (HbA1c < 5.7) (*n* = 252)	Case (HbA1c ≥ 5.7) (*n* = 219)	*p*value
Chronic lung disease, *n* (%)	0	3 (1.4)	0.100
Asthma, *n* (%)	0	3 (1.4)	0.100
CKD, *n* (%)	4 (1.6)	8 (3.7)	0.240
CAD, *n* (%)	2 (0.8)	10 (4.6)	0.015
Congenital heart disease, *n* (%)	4 (1.6)	2 (0.9)	0.690
Cerebrovascular disease, *n* (%)	1 (0.4)	2 (0.9)	0.600
Cirrhosis, *n* (%)	1 (0.4)	0	1.000
HTN, *n* (%)	36 (14.3)	95 (43.4)	< 0.001
DLP, *n* (%)	24 (9.5)	63 (28.8)	< 0.001
DM, *n* (%)	5 (2)	64 (29.2)	< 0.001
Immunocompromised host, *n* (%)	6 (2.4)	1 (0.5)	0.129
Immunocompromised HIV, *n* (%)	10 (4)	2 (0.9)	0.042
Pregnancy, *n* (%)	2 (0.8)	0	0.501
Allergy, *n* (%)	17 (6.7)	12 (5.5)	0.568
Gout, *n* (%)	1 (0.4)	5 (2.3)	0.101
Thyroid, *n* (%)	9 (3.6)	5 (2.3)	0.412

*Note:* Data showed *n* (%) and were analyzed by chi‐square or Fisher′s exact test (count less than 5). *p* value < 0.05 was considered statistically significant.

Abbreviations: CAD, coronary artery disease; CKD, chronic kidney disease; DLP, dyslipidemia; DM, diabetes mellitus; HIV, human immunodeficiency virus; HTN, hypertension.

Hypertension was markedly more common among hyperglycemic participants than normoglycemic controls (43.4% vs. 14.3%, *p* < 0.001), as was hyperlipidemia (28.8% vs. 9.5%, *p* < 0.001) (Table 2). Diabetes mellitus was also substantially more prevalent in the hyperglycemia group (29.2% vs. 2.0%, *p* < 0.001), whereas coronary artery disease occurred more frequently in hyperglycemic individuals (4.6% vs. 0.8%, *p* = 0.015). No statistically significant differences were observed for chronic kidney disease, chronic lung disease, asthma, cerebrovascular disease, thyroid disease, or immunocompromised status, although HIV‐associated immunocompromise was more frequent in the normoglycemic group (*p* = 0.042) (Table 2). Given these clinically meaningful differences, age, BMI, and underlying diseases were included as covariates in multivariable regression analyses to reduce confounding.

### 3.3. Association Between Innate Immune Gene Polymorphisms and Hyperglycemia

**Figure 2:**
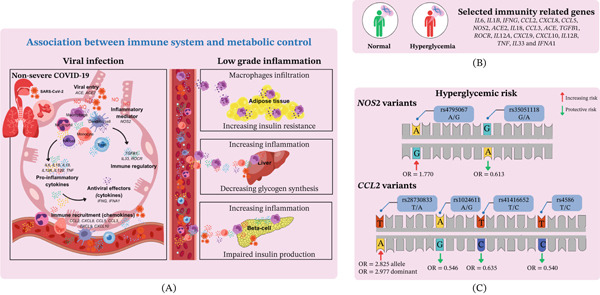
Association among the immune system, metabolic control, and genetic polymorphisms in post‐COVID‐19 hyperglycemia. (A) Upon nonsevere SARS‐CoV‐2 infection, immune cells such as neutrophils, macrophages, monocytes, dendritic cells, and T cells are activated, leading to the release of nitric oxide (NO) and a range of immune mediators. These include proinflammatory cytokines (e.g., IL‐6, IL‐1B, IL‐18, IL12A, IL12B, and TNF), chemokines that promote immune cell recruitment (e.g., CCL2, CXCL8, CCL5, CCL3, CXCL9, and CXCL10), and antiviral cytokines (e.g., IFNG and IFNA1). *NOS2* acts as a key inflammatory mediator through NO production, whereas other genes such as *TGFB1*, *IL33*, and *ROCR* contribute to immune regulation. Low‐grade inflammation persists postinfection, contributing to insulin resistance in adipose tissue, reduced glycogen synthesis in the liver, and impaired insulin production in pancreatic *β*‐cells. (B) Based on HbA1c levels, individuals recovering from COVID‐19 were classified as normoglycemic or hyperglycemic. Twenty immunity‐related genes were selected for SNP association analysis. (C) Variants in the *NOS2* and *CCL2* genes showed significant associations with hyperglycemic risk. In NOS2, SNP rs4795067 (AG genotype) increased risk (OR = 1.770), whereas rs35051118 (GA genotype) showed a protective effect (OR = 0.613). In *CCL2*, rs28730833 (TA genotype and A allele) conferred the highest risk (OR = 2.977 and OR = 2.825, respectively), whereas heterozygous genotypes of rs1024611, rs41416652, and rs4586 were associated with reduced risk of hyperglycemia.

To investigate whether innate immune genetic variation was associated with hyperglycemia, candidate polymorphisms in *NOS2* and *CCL2* were evaluated using binary logistic regression analysis (Tables 3 and 4). Within the *NOS2* gene, the rs4795067 polymorphism demonstrated a significant association with hyperglycemia following adjustment for age, BMI, and underlying diseases (Table 3). Individuals carrying the heterozygous AG genotype had significantly increased odds of hyperglycemia compared with the AA reference genotype (adjusted OR = 1.770, 95% CI: 1.137–2.756, *p* = 0.011) (Table 4). In contrast, the rs35051118 GA genotype was associated with reduced odds of hyperglycemia (adjusted OR = 0.613, 95% CI: 0.386–0.975, *p* = 0.039), suggesting a potential protective effect. Analysis of *CCL2* polymorphisms *F* *D* *J* *G* identified several significant associations. Among these, rs28730833 demonstrated the strongest association with hyperglycemia (Tables 3 and 4). Individuals carrying the TA genotype had nearly threefold increased odds of hyperglycemia compared with the TT reference genotype (adjusted OR = 2.977, 95% CI: 1.349–6.572, *p* = 0.007). Similarly, carriage of the minor A allele was associated with increased risk (adjusted OR = 2.825, 95% CI: 1.307–6.105, *p* = 0.008). Conversely, several *CCL2* variants were associated with reduced odds of hyperglycemia. The rs1024611 AG genotype showed a protective association (adjusted OR = 0.546, 95% CI: 0.334–0.891, *p* = 0.015), as did rs4586 TC (adjusted OR = 0.540, 95% CI: 0.330–0.884, *p* = 0.014) and rs41416652 TC (adjusted OR = 0.635, 95% CI: 0.410–0.984, *p* = 0.042) (Table 4). Collectively, these findings suggest that both susceptibility and protective variants within innate immune regulatory pathways may contribute to heterogeneity in glycemic outcomes following COVID‐19 (Figure 2).

**Table 3:** Frequency of innate immune gene polymorphisms in hyperglycemic patients.

Genes	Chromosome: Position SNP	Allelic alteration	Genotype and allele	Control (HbA1c < 5.7), *n* (%)	Case (HbA1c ≥ 5.7), *n* (%)	*p*value
*NOS2*	Chr17: 27779649	A > G	AA	153 (60.7)	123 (56.2)	**0.004**
	rs4795067		AG	77 (30.6)	90 (41.1)	
			GG	22 (8.7)	6 (2.7)	
			A allele	383 (76)	336 (76.7)	0.795
			G allele	121 (24)	102 (23.3)	
	Chr17: 27793946	G > A	GG	160 (63.7)	156 (71.2)	0.195
	rs35051118		GA	82 (32.7)	55 (25.1)	
			AA	9 (3.6)	8 (3.7)	
			G allele	402 (80.1)	367 (83.8)	0.141
			A allele	100 (19.9)	71 (16.2)	
*CCL2*	Chr17: 34252769	A > G	AA	62 (24.6)	71 (32.4)	0.151
	rs1024611		AG	132 (52.4)	99 (45.2)	
			GG	58 (23)	49 (22.4)	
			A allele	256 (50.8)	214 (55)	0.195
			G allele	248 (49.2)	197 (45)	
	Chr17: 34255475	T > A	TT	236 (94)	191 (88)	**0.022**
	rs28730833		TA	15 (6)	26 (12)	
			T allele	487 (97)	408 (94)	**0.025**
			A allele	15 (3)	26 (6)	
	Chr17: 34256250	T > C	TT	61 (24.2)	69 (31.5)	0.140
	rs4586		TC	136 (54)	100 (45.7)	
			CC	55 (21.8)	50 (22.8)	
			T allele	258 (51.2)	238 (54.3)	0.335
			C allele	246 (48.8)	200 (45.7)	
	Chr17: 34258842	T > C	TT	112 (44.6)	108 (49.3)	0.194
	rs41416652		TC	118 (47)	86 (39.3)	
			CC	21 (8.4)	25 (11.4)	
			T allele	342 (68.1)	302 (68.9)	0.787
			C allele	160 (31.9)	136 (31.1)	

*Note:* Data showed frequency (*n*) and percentage (%). *p* value was analyzed by the chi‐square test. Bold *p* values indicate significant differences between SNP frequencies and patient groups (*p* value less than 0.05).

**Table 4:** Logistic regression analysis between innate immune gene polymorphisms and hyperglycemic patients.

Genes	Chromosome: Position SNP	Allelic alteration	Genotype and allele	Crude results			Adjusted results		
				OR	95% CI	*p*value	OR	95% CI	*p*value
*NOS2*	Chr17: 27779649	A > G	AA	Ref	—	—	Ref	—	—
	rs4795067		AG	1.454	0.988, 2.139	0.057	**1.770**	**1.137, 2.756**	**0.011**
			GG	**0.339**	**0.133, 0.863**	**0.023**	0.426	0.147, 1.236	0.116
			A allele	Ref	—	—	Ref	—	—
			G allele	0.961	0.711, 1.299	0.795	1.139	0.807, 1.608	0.459
	Chr17: 27793946	G > A	GG	Ref	—	—	Ref	—	—
	rs35051118		GA	0.688	0.458, 1.033	0.071	**0.613**	**0.386, 0.975**	**0.039**
			AA	0.912	0.343, 2.423	0.853	0.885	0.285, 2.744	0.832
			G allele	Ref	—	—	Ref	—	—
			A allele	0.778	0.556, 1.088	0.142	0.719	0.491, 1.053	0.090
*CCL2*	Chr17: 34252769	A > G	AA	Ref	—	—	Ref	—	—
	rs1024611		AG	0.655	0.426, 1.006	0.053	**0.546**	**0.334, 0.891**	**0.015**
			GG	0.738	0.443, 1.229	0.243	0.617	0.343, 1.107	0.106
			A allele	Ref	—	—	Ref	—	—
			G allele	0.844	0.653, 1.091	0.195	0.766	0.572, 1.026	0.074
	Chr17: 34255475	T > A	TT	Ref	—	—	Ref	—	—
	rs28730833		TA	**2.142**	**1.103, 4.158**	**0.024**	**2.977**	**1.349, 6.572**	**0.007**
			T allele	Ref	—	—	Ref	—	—
			A allele	**2.069**	**1.081, 3.959**	**0.028**	**2.825**	**1.307, 6.105**	**0.008**
	Chr17: 34256250	T > C	TT	Ref	—	—	Ref	—	—
	rs4586		TC	0.650	0.423, 1.000	0.050	**0.540**	**0.330, 0.884**	**0.014**
			CC	0.804	0.480, 1.345	0.406	0.649	0.360, 1.170	0.151
			T allele	Ref	—	—	Ref	—	—
			C allele	0.881	0.682, 1.139	0.335	0.789	0.589, 1.057	0.112
	Chr17: 34258842	T > C	TT	Ref	—	—	Ref	—	—
	rs41416652		TC	0.756	0.515, 1.109	0.152	**0.635**	**0.410, 0.984**	**0.042**
			CC	1.235	0.653, 2.336	0.517	0.876	0.424, 1.809	0.720
			T allele	Ref	—	—	Ref	—	—
			C allele	0.963	0.730, 1.269	0.787	0.814	0.595, 1.113	0.197

*Note:* All data were analyzed by binary logistic regression. Reference categorically was control patients with homozygous wildtype and major allele. Data were adjusted for age, body mass index (BMI), and underlying disease. Bold *p* value indicates significance (*p* value < 0.05).

Abbreviations: OR (95% CI), odds ratio (95% confidence interval); Ref, reference.

### 3.4. Genotype–Phenotype Associations

To further explore the potential biological relevance of significant genetic variants, genotype–phenotype analyses were performed among hyperglycemic participants (Tables 5 and 6). For *NOS2*, rs4795067 did not demonstrate significant associations with HbA1c, CRP, creatinine, or antibody responses (Table 5). However, carriers of the G allele exhibited significantly lower IL‐6 levels at 1 month compared with A allele carriers (*p* = 0.031), suggesting a potential influence on inflammatory signaling rather than direct metabolic severity. Similarly, rs35051118 was associated with lower CRP levels (*p* = 0.013) and reduced creatinine levels (*p* = 0.018), indicating possible effects on inflammatory or systemic physiological responses. Among *CCL2* variants, rs1024611 was associated with higher HbA1c levels at 3 months (*p* = 0.042), suggesting a relationship with persistent glycemic dysregulation (Table 6). The rs28730833 variant demonstrated significant associations with reduced anti‐RBD IgG levels at both 1 month (*p* = 0.025) and 3 months (*p* = 0.006), potentially indicating altered humoral immune responses. The rs41416652 variant showed broader phenotype associations, including higher CRP levels (*p* = 0.037), elevated HbA1c at 1 month (*p* = 0.014), and increased IL‐6 levels at 3 months (*p* = 0.022), suggesting a potential role in sustained inflammatory‐metabolic interactions (Table 6). Taken together, these genotype–phenotype findings provide preliminary evidence that specific *NOS2* and *CCL2* variants may influence inflammatory and metabolic recovery trajectories following COVID‐19.

**Table 5:** Clinical outcomes of *NOS2* gene polymorphisms in post‐COVID‐19 patients with hyperglycemia.

**Chr17: 27779649 (A > G)**	**Genotype**				**Allele**		
**Clinical characteristics**	**AA**	**AG**	**GG**	*p* **value**	**A allele**	**G allele**	*p* **value**

CRP follow‐up 1 month (mg/dL)	1.30 (0.60, 4.63) (*n* = 9)	1.84 (0.62, 3.31) (*n* = 5)	—	0.893	1.30 (0.60, 3.12) (*n* = 23)	1.84 (0.62, 3.31) (*n* = 5)	0.903
CRP follow‐up 3 months (mg/dL)	1.63 (0.73, 3.46) (*n* = 122)	1.80 (1.05, 3.39) (*n* = 90)	2.40 (1.01, 4.37) (*n* = 6)	0.602	1.69 (0.77, 3.45) (*n* = 334)	1.88 (1.10, 3.50) (*n* = 102)	0.356
HbA1c follow‐up 1 month (%)	6.90 (6.35, 8.50) (*n* = 9)	6.80 (6.30, 9.35) (*n* = 5)	—	0.738	6.8 (6.3, 8.0) (*n* = 23)	6.9 (6.3, 9.4) (*n* = 5)	0.763
HbA1c follow‐up 3 months (%)	6.1 (5.8, 6.6) (*n* = 123)	6.1 (5.8, 6.5) (*n* = 90)	6.0 (6.0, 6.9) (*n* = 6)	0.968	6.1 (5.8, 6.6) (*n* = 336)	6.1 (5.8, 6.4) (*n* = 102)	0.820
Cr follow‐up 1 month (mg/dL)	0.75 ± 0.20 (*n* = 9)	0.86 ± 0.23 (*n* = 5)	—	0.355	0.77 ± 0.21 (*n* = 23)	0.86 ± 0.23 (*n* = 5)	0.394
Cr follow‐up 3 months (mg/dL)	0.79 (0.65, 1.00) (*n* = 122)	0.72 (0.61, 0.94) (*n* = 90)	0.85 (0.75, 1.05) (*n* = 6)	0.184	0.75 (0.62, 0.98) (*n* = 334)	0.73 (0.61, 0.96) (*n* = 102)	0.418
Anti‐RBD IgG level follow‐up 1 month (AU/mL)	10,245.8 (4226.3, 18,332.5) (*n* = 122)	7504.3 (3769.9, 15,820.9) (*n* = 90)	10,512.4 (5842.5, 25,400.8) (*n* = 6)	0.680	8989.6 (4160.9, 18,259.6) (*n* = 334)	7761.0 (4136.7, 17,900.7) (*n* = 102)	0.877
Anti‐RBD IgG level follow‐up 3 months (AU/mL)	8989.6 (4226.3, 17,430.4) (*n* = 122)	7504.3 (3769.9, 14,530.7) (*n* = 90)	10,512.4 (5842.5, 25,400.8) (*n* = 6)	0.642	8449.4 (4160.9, 17,175.0) (*n* = 334)	7761.0 (4136.7, 14,827.5) (*n* = 102)	0.963
IL‐6 follow‐up 1 month (pg/mL)	1.50 (1.50, 2.00) (*n* = 122)	1.50 (1.50, 1.50) (*n* = 90)	1.50 (1.50, 1.50) (*n* = 6)	0.057	1.50 (1.50, 1.78) (*n* = 334)	1.50 (1.50, 1.50) (*n* = 102)	0.031
IL‐6 follow‐up 3 months (pg/mL)	1.57 (1.50, 2.91) (*n* = 8)	1.54 (1.50, 2.05) (*n* = 5)	—	0.878	1.54 (1.50, 2.14) (*n* = 21)	1.54 (1.50, 2.05) (*n* = 5)	0.891

**Chr17: 27793946 (G > A)**	**Genotype**				**Allele**		
**Clinical characteristics**	**GG**	**GA**	**AA**	**p value**	**G allele**	**A allele**	**pvalue**

CRP follow‐up 1 month (mg/dL)	1.84 (0.60, 3.31) (*n* = 9)	—	—	—	1.84 (0.60, 3.31) (*n* = 21)	0.82 (0.60, 2.27) (*n* = 7)	0.361
CRP follow‐up 3 months (mg/dL)	1.79 (1.03, 3.74) (*n* = 155)	1.72 (0.67, 2.86) (*n* = 55)	0.66 (0.60, 0.86) (*n* = 8)	0.437	1.79 (0.98, 3.66) (*n* = 365)	1.51 (0.60, 2.41) (*n* = 71)	0.013
HbA1c follow‐up 1 month (%)	7.93 ± 2.40 (*n* = 9)	7.57 ± 1.24 (*n* = 3)	—	0.578	6.8 (6.3, 8.5) (*n* = 21)	6.7 (5.9, 6.9) (*n* = 7)	0.287
HbA1c follow‐up 3 months (%)	6.1 (5.8, 6.6) (*n* = 156)	6.0 (5.8, 6.6) (*n* = 55)	6.25 (5.83, 6.63) (*n* = 8)	0.279	6.1 (5.8, 6.6) (*n* = 367)	6.1 (5.8, 6.6) (*n* = 71)	0.429
Cr follow‐up 1 month (mg/dL)	0.80 ± 0.20 (*n* = 9)	0.81 ± 0.30 (*n* = 3)	—	0.988	0.80 ± 0.20 (*n* = 21)	0.74 ± 0.24 (*n* = 7)	0.486
Cr follow‐up 3 months (mg/dL)	0.74 (0.61, 0.95) (*n* = 155)	0.80 (0.65, 1.03) (*n* = 55)	0.86 (0.60, 1.19) (*n* = 8)	0.065	0.74 (0.61, 0.95) (*n* = 365)	0.82 (0.65, 1.07) (*n* = 71)	0.018
Anti‐RBD IgG level follow‐up 1 month (AU/mL)	8303.6 (4392.3, 17,087.3) (*n* = 156)	9203.2 (3086.7, 23,347.5) (*n* = 54)	24,317 (6375, 44,866) (*n* = 8)	0.220	8314.3 (4223.7, 17,388.0) (*n* = 366)	12,232.5 (3739.3, 29,777.3) (*n* = 70)	0.282
Anti‐RBD IgG level follow‐up 3 months (AU/mL)	8094.3 (4387.2, 15,918.5) (*n* = 155)	7147.4 (3107.1, 22,705.9) (*n* = 55)	16,216 (6375, 37,485) (*n* = 8)	0.291	8094.3 (4221.9, 16,031.6) (*n* = 365)	10,706.6 (3933.7, 24,054.5) (*n* = 71)	0.353
IL‐6 follow‐up 1 month (pg/mL)	1.50 (1.50, 1.53) (*n* = 156)	1.50 (1.50, 2.12) (*n* = 54)	1.50 (1.50, 2.25) (*n* = 8)	0.228	1.50 (1.50, 1.62) (*n* = 366)	1.50 (1.50, 2.12) (*n* = 70)	0.190
IL‐6 follow‐up 3 months (pg/mL)	1.57 (1.50, 1.83) (*n* = 8)	—	—	—	1.63 (1.50, 1.87) (*n* = 19)	1.50 (1.50, 3.25) (*n* = 7)	0.715

*Note:* Data showed mean ± SD or median (Q1, Q3) according to data distribution.

Abbreviations: AU/mL, arbitrary concentration units; BMI, body mass index; Cr, creatinine; CRP, C‐reactive protein; IL‐6, interleukin‐6; Nab, neutralizing antibody.

**Table 6:** Clinical outcomes of *CCL2* gene polymorphisms in post‐COVID‐19 patients with hyperglycemia.

**Chr17: 34252769 (A > G)**	**Genotype**				**Allele**		
**Clinical characteristics**	**AA**	**AG**	**GG**	*p* **value**	**A allele**	**G allele**	*p* **value**

CRP follow‐up 1 month (mg/dL)	1.09 ± 0.662 (*n* = 3)	2.26 ± 2.08 (*n* = 8)	—	0.465	1.06 (0.60, 2.35) (*n* = 14)	2.43 (0.62, 4.29) (*n* = 14)	0.209
CRP follow‐up 3 months (mg/dL)	1.80 (0.72, 3.82) (*n* = 71)	1.63 (0.81, 3.28) (*n* = 99)	2.01 (0.97, 3.34) (*n* = 48)	0.737	1.66 (0.77, 3.66) (*n* = 241)	1.79 (0.93, 3.34) (*n* = 195)	0.665
HbA1c follow‐up 1 month (%)	6.47 ± 0.49 (*n* = 3)	7.05 ± 1.00 (*n* = 8)	10.30 ± 3.04 (*n* = 3)	0.129	6.7 (6.3, 7.0) (*n* = 14)	7.1 (6.5, 11.9) (*n* = 14)	0.065
HbA1c follow‐up 3 months (%)	6.0 (5.8, 6.5) (*n* = 71)	6.1 (5.8, 6.7) (*n* = 99)	6.3 (6.0, 6.9) (*n* = 49)	0.042	6.0 (5.8, 6.5) (*n* = 241)	6.1 (5.8, 6.7) (*n* = 197)	0.021
Cr follow‐up 1 month (mg/dL)	0.76 ± 0.22 (*n* = 3)	0.79 ± 0.23 (*n* = 8)	0.81 ± 0.24 (*n* = 3)	0.960	0.78 ± 0.21 (*n* = 14)	0.80 ± 0.22 (*n* = 14)	0.777
Cr follow‐up 3 months (mg/dL)	0.73 (0.61, 0.95) (*n* = 71)	0.76 (0.61, 0.98) (*n* = 99)	0.75 (0.64, 1.01) (*n* = 48)	0.812	0.74 (0.61, 0.96) (*n* = 241)	0.75 (0.63, 0.98) (*n* = 195)	0.502
Anti‐RBD IgG level follow‐up 1 month (AU/mL)	7760.95 (3344.70, 16,675.90) (*n* = 70)	9749.0 (3849.1, 23,111.8) (*n* = 99)	8085.7 (5200.3, 22,916.2) (*n* = 49)	0.457	8449.4 (3620.0, 17,557.6) (*n* = 239)	9088.8 (4519.6, 22,908.9) (*n* = 197)	0.216
Anti‐RBD IgG level follow‐up 3 months (AU/mL)	7609.50 (3449.80, 16,031.60) (*n* = 71)	8890.4 (3849.1, 18,259.6) (*n* = 99)	7890.7 (5168.5, 21,235.5) (*n* = 48)	0.472	8094.3 (3720.0, 16,526.3) (*n* = 241)	8449.4 (4519.6, 18,259.6) (*n* = 195)	0.213
IL‐6 follow‐up 1 month (pg/mL)	1.50 (1.50, 1.50) (*n* = 70)	1.50 (1.50, 1.91) (*n* = 99)	1.50 (1.50, 1.64) (*n* = 49)	0.407	1.50 (1.50, 1.68) (*n* = 239)	1.50 (1.50, 1.71) (*n* = 197)	0.275
IL‐6 follow‐up 3 months (pg/mL)	—	1.66 (1.50, 2.91) (*n* = 8)	—	—	1.50 (1.50, 1.73) (*n* = 14)	1.66 (1.51, 2.41) (*n* = 12)	0.116

**Chr17: 34255475 (T > A)**	**Genotype**				**Allele**		
**Clinical characteristics**	**TT**	**TA**	**AA**	**p value**	**T allele**	**A allele**	**p value**

CRP follow‐up 1 month (mg/dL)	1.06 (0.60, 2.99) (*n* = 12)	—	—	—	1.30 (0.60, 3.12) (*n* = 26)	—	—
CRP follow‐up 3 months (mg/dL)	1.69 (0.81, 3.45) (*n* = 190)	2.13 (1.05, 3.54) (*n* = 26)	—	0.406	1.71 (0.82, 3.45) (*n* = 406)	2.13 (1.05, 3.54) (*n* = 26)	0.421
HbA1c follow‐up 1 month (%)	6.80 (6.38, 8.75) (*n* = 12)	—	—	—	6.8 (6.3, 8.3) (*n* = 26)	—	—
HbA1c follow‐up 3 months (%)	6.1 (5.8, 6.6) (*n* = 191)	6.0 (5.8, 6.8) (*n* = 26)	—	0.799	6.1 (5.8, 6.6) (*n* = 408)	6.0 (5.8, 6.8) (*n* = 26)	0.805
Cr follow‐up 1 month (mg/dL)	0.77 ± 0.22 (*n* = 12)	—	—	—	0.78 ± 0.21 (*n* = 26)	—	—
Cr follow‐up 3 months (mg/dL)	0.74 (0.61, 0.97) (*n* = 190)	0.79 (0.66, 1.01) (*n* = 26)	—	0.429	0.74 (0.61, 0.98) (*n* = 406)	0.79 (0.66, 1.01) (*n* = 26)	0.443
Anti‐RBD IgG level follow‐up 1 month (AU/mL)	9635.5 (4407.7, 18,551.3) (*n* = 191)	5015.5 (2622.9, 11,597.3) (*n* = 25)	—	0.025	8890.4 (4228.9, 18,432.8) (*n* = 407)	5015.5 (2622.9, 11,597.3) (*n* = 25)	0.030
Anti‐RBD IgG level follow‐up 3 months (AU/mL)	8989.6 (4389.8, 18,190.5) (*n* = 190)	5169.4 (2638.0, 11,110.3) (*n* = 26)	—	0.006	8559.4 (4228.9, 17,430.4) (*n* = 406)	5169.4 (2638.0, 11,110.3) (*n* = 26)	0.008
IL‐6 follow‐up 1 month (pg/mL)	1.50 (1.50, 1.75) (*n* = 191)	1.50 (1.50, 1.50) (*n* = 25)	—	0.419	1.50 (1.50, 1.71) (*n* = 407)	1.50 (1.50, 1.50) (*n* = 25)	0.434
IL‐6 follow‐up 3 months (pg/mL)	1.50 (1.50, 1.87) (*n* = 11)	—	—	—	1.52 (1.50, 1.87) (*n* = 24)	—	0.105

**Chr17: 34256250 (T > C)**	**Genotype**				**Allele**		
**Clinical characteristics**	**TT**	**TC**	**CC**	**p value**	**T allele**	**C allele**	**p value**

CRP follow‐up 1 month (mg/dL)	1.09 ± 0.66 (*n* = 3)	2.26 ± 2.08 (*n* = 8)	4.11 ± 4.07 (*n* = 3)	0.404	1.06 (0.60, 2.35) (*n* = 14)	2.43 (0.62, 4.29) (*n* = 14)	0.209
CRP follow‐up 3 months (mg/dL)	1.80 (0.73, 3.78) (*n* = 69)	1.60 (0.81, 3.27) (*n* = 100)	2.04 (1.00, 3.36) (*n* = 49)	0.653	1.66 (0.77, 3.66) (*n* = 238)	1.79 (0.92, 3.34) (*n* = 198)	0.608
HbA1c follow‐up 1 month (%)	6.47 ± 0.49 (*n* = 3)	7.05 ± 1.00 (*n* = 8)	10.30 ± 3.04 (*n* = 3)	0.129	6.7 (6.3, 7.0) (*n* = 14)	7.1 (6.5, 11.9) (*n* = 14)	0.065
HbA1c follow‐up 3 months (%)	6.0 (5.8, 6.5) (*n* = 69)	6.1 (5.8, 6.7) (*n* = 100)	6.3 (6.0, 6.8) (*n* = 50)	0.065	6.0 (5.8, 6.5) (*n* = 238)	6.1 (5.8, 6.6) (*n* = 200)	0.021

**Chr17: 34256250 (T > C)**	**Genotype**				**Allele**		
**Clinical characteristics**	**TT**	**TC**	**CC**	**p value**	**T allele**	**C allele**	**p value**

Cr follow‐up 1 month (mg/dL)	0.76 ± 0.22 (*n* = 3)	0.79 ± 0.23 (*n* = 8)	0.81 ± 0.24 (*n* = 3)	0.960	0.78 ± 0.21 (*n* = 14)	0.80 ± 0.22 (*n* = 14)	0.777
Cr follow‐up 3 months (mg/dL)	0.74 (0.61, 0.96) (*n* = 69)	0.76 (0.62, 0.98) (*n* = 100)	0.74 (0.63, 1.00) (*n* = 49)	0.877	0.74 (0.61, 0.96) (*n* = 238)	0.75 (0.63, 0.98) (*n* = 198)	0.597
Anti‐RBD IgG level follow‐up 1 month (AU/mL)	7955.8 (3542.3, 16,975.1) (*n* = 68)	9692.3 (3870.3, 22,735.5) (*n* = 100)	7890.7 (4999.1, 22,811.1) (*n* = 50)	0.637	8577.7 (3819.9, 17,557.6) (*n* = 236)	8724.9 (4516.2, 22,705.9) (*n* = 200)	0.363
Anti‐RBD IgG level follow‐up 3 months (AU/mL)	7912.4 (3634.9, 16,279.0) (*n* = 69)	8724.9 (3870.3, 18,041.7) (*n* = 100)	7695.7 (4861.4, 19,765.1) (*n* = 49)	0.652	8168.5 (3819.9, 16,675.9) (*n* = 238)	8168.5 (4491.3, 17,605.9) (*n* = 198)	0.355
IL‐6 follow‐up 1 month (pg/mL)	1.50 (1.50, 1.50) (*n* = 68)	1.50 (1.50, 1.74) (*n* = 100)	1.50 (1.50, 1.78) (*n* = 50)	0.504	1.50 (1.50, 1.67) (*n* = 236)	1.50 (1.50, 1.71) (*n* = 200)	0.269
IL‐6 follow‐up 3 months (pg/mL)	—	1.66 (1.50, 2.91) (*n* = 8)	—	—	1.50 (1.50, 1.73) (*n* = 14)	1.66 (1.51, 2.41) (*n* = 12)	0.116
**Chr17: 34258842 (T > C)**	**Genotype**				**Allele**		
**Clinical characteristics**	**TT**	**TC**	**CC**	**p value**	**T allele**	**C allele**	**p value**
CRP follow‐up 1 month (mg/dL)	0.71 (0.60, 2.25) (*n* = 6)	1.79 (0.62, 3.61) (*n* = 6)	—	0.289	0.82 (0.60, 2.35) (*n* = 18)	2.85 (1.13, 7.16) (*n* = 10)	0.037
CRP follow‐up 3 months (mg/dL)	1.56 (0.75, 3.47) (*n* = 108)	1.73 (1.03, 3.39) (*n* = 86)	1.90 (0.88, 3.60) (*n* = 24)	0.594	1.67 (0.81, 3.45) (*n* = 302)	1.84 (0.93, 3.39) (*n* = 134)	0.447
HbA1c follow‐up 1 month (%)	6.5 (6.2, 6.9) (*n* = 6)	6.85 (6.48, 8.25) (*n* = 6)	—	0.228	6.7 (6.3, 7.0) (*n* = 18)	8.5 (6.8, 12.0) (*n* = 10)	0.014
HbA1c follow‐up 3 months (%)	6.0 (5.8, 6.4) (*n* = 108)	6.1 (5.8, 6.9) (*n* = 86)	6.0 (5.8, 6.6) (*n* = 25)	0.373	6.1 (5.8, 6.5) (*n* = 302)	6.1 (5.8, 6.6) (*n* = 136)	0.355
Cr follow‐up 1 month (mg/dL)	0.86 ± 0.22 (*n* = 6)	0.73 ± 0.21 (*n* = 6)	—	0.313	0.82 ± 0.21 (*n* = 18)	0.74 ± 0.20 (*n* = 10)	0.319
Cr follow‐up 3 months (mg/dL)	0.79 (0.61, 0.99) (*n* = 108)	0.75 (0.61, 0.99) (*n* = 86)	0.74 (0.65, 0.86) (*n* = 24)	0.903	0.75 (0.61, 0.98) (*n* = 302)	0.74 (0.62, 0.94) (*n* = 134)	0.694
Anti‐RBD IgG level follow‐up 1 month (AU/mL)	7912.4 (3819.9, 16,031.6) (*n* = 107)	9949.9 (4110.9, 26,577.2) (*n* = 86)	10,258.6 (4861.4, 19,765.1) (*n* = 25)	0.461	8157.8 (3827.2, 17,515.2) (*n* = 300)	10,258.6 (4586.0, 23,122.8) (*n* = 136)	0.229
Anti‐RBD IgG level follow‐up 3 months (AU/mL)	7804.0 (3827.2, 15,670.1) (*n* = 108)	8724.9 (4110.9, 24,280.3) (*n* = 86)	9673.7 (4723.7, 16,070.6) (*n* = 24)	0.489	8042.4 (3841.8, 16,155.3) (*n* = 302)	9088.8 (4532.2, 21,881.3) (*n* = 134)	0.247
IL‐6 follow‐up 1 month (pg/mL)	1.50 (1.50, 1.64) (*n* = 107)	1.50 (1.50, 1.70) (*n* = 86)	1.50 (1.50, 2.62) (*n* = 25)	0.312	1.50 (1.50, 1.65) (*n* = 300)	1.50 (1.50, 1.98) (*n* = 136)	0.194
IL‐6 follow‐up 3 months (pg/mL)	1.50 (1.50, 1.55) (*n* = 6)	1.75 (1.53, 4.91) (*n* = 6)	—	0.033	1.50 (1.50, 1.69) (*n* = 18)	2.14 (1.56, 3.04) (*n* = 8)	0.022

*Note:* Data showed mean ± SD or median (Q1, Q3), as appropriate based on the data distribution.

Abbreviations: AU/mL, arbitrary concentration units; Cr, creatinine; CRP, C‐reactive protein; IL‐6, interleukin‐6; Nab, neutralizing antibody.

## 4. Discussion

This study investigated the association between innate immune genetic polymorphisms and hyperglycemia among individuals recovering from COVID‐19. Our findings demonstrate that post‐COVID participants with hyperglycemia exhibited a less favorable metabolic profile, characterized by older age, higher BMI, greater cardiometabolic comorbidity burden, and evidence of persistent low‐grade inflammation compared with normoglycemic individuals. More importantly, genetic association analysis identified significant polymorphisms within *NOS2* and *CCL2* that were associated with differential odds of hyperglycemia, suggesting that host innate immune genetic variation may contribute to interindividual heterogeneity in metabolic outcomes following SARS‐CoV‐2 infection.

A central finding of this study was the association between *NOS2* polymorphisms and hyperglycemia. *NOS2* encodes iNOS, a key enzyme responsible for nitric oxide production during inflammatory activation [22]. Although nitric oxide plays essential roles in antimicrobial defense and immune regulation [23], excessive iNOS activation has been implicated in oxidative stress, mitochondrial dysfunction, pancreatic *β*‐cell injury, and impaired insulin receptor signaling [24], all of which contribute to metabolic dysregulation and insulin resistance. Experimental studies have shown that excessive nitric oxide production may impair insulin‐mediated glucose uptake and promote inflammatory‐metabolic dysfunction, particularly under chronic inflammatory conditions [16, 17]. The observed association between *NOS2* rs4795067 and increased odds of hyperglycemia in our cohort may therefore reflect altered inflammatory or oxidative responses influencing glucose homeostasis during post‐COVID recovery. Conversely, the protective association observed with rs35051118 suggests that genetic variation within this pathway may exert differential regulatory effects on immune‐metabolic balance.

The strongest genetic association identified in this study involved *CCL2* rs28730833, which was associated with substantially increased odds of hyperglycemia after adjustment for age, BMI, and underlying comorbidities. *CCL2*, also known as MCP‐1, is a critical chemokine regulating monocyte recruitment, macrophage activation, and sustained inflammatory signaling [25]. Dysregulated MCP‐1 signaling has been strongly implicated in obesity‐associated inflammation, adipose tissue macrophage infiltration, insulin resistance, and progression toward metabolic disease [18, 19]. In the context of viral infection, prolonged activation of chemokine‐mediated inflammatory pathways may contribute to persistent immune dysregulation and metabolic stress [26]. The observed association between *CCL2* variants and hyperglycemia supports the hypothesis that inflammatory chemokine signaling may represent an important biological link between postinfectious immune activation and altered glucose regulation.

Interestingly, several *CCL2* polymorphisms, including rs1024611, rs4586, and rs41416652, were associated with reduced odds of hyperglycemia, suggesting that not all immune genetic variants confer increased metabolic risk. Rather, these findings support the concept that host genetic architecture may differentially modulate inflammatory susceptibility, potentially conferring either vulnerability or relative protection depending on the specific regulatory variant involved [27]. The genotype–phenotype analyses further support this interpretation, as certain *CCL2* variants were associated with differences in inflammatory markers, glycemic measurements, and humoral immune responses. Although these findings remain exploratory, they provide preliminary biological plausibility for immune‐metabolic heterogeneity following COVID‐19.

Our findings are broadly consistent with previous literature highlighting the role of chronic inflammation in metabolic dysfunction. Persistent low‐grade inflammation is a well‐established driver of insulin resistance through cytokine‐mediated impairment of insulin signaling, oxidative injury, and immune cell infiltration into metabolically active tissues [9, 10]. COVID‐19 is characterized by substantial immune activation during acute infection, and persistent inflammatory dysregulation has been reported in subsets of recovering patients [3, 4, 6]. Our findings extend this concept by suggesting that host genetic variation within innate immune pathways may partially explain why some individuals demonstrate hyperglycemia during recovery, whereas others maintain metabolic stability.

However, interpretation of these findings requires caution. Participants with hyperglycemia in this cohort had a significantly higher burden of baseline cardiometabolic comorbidities, including hypertension, dyslipidemia, coronary artery disease, and pre‐existing diabetes mellitus, all of which independently increase the likelihood of impaired glucose regulation [28, 29]. Although multivariable models adjusted for age, BMI, and underlying diseases, residual confounding cannot be excluded. Therefore, the identified polymorphisms should not be interpreted as independent causal determinants of hyperglycemia, but rather as candidate susceptibility factors within a broader multifactorial metabolic context.

Another important limitation is the absence of preinfection glycemic measurements. Consequently, it cannot be determined whether hyperglycemia identified during follow‐up developed after SARS‐CoV‐2 infection or represented pre‐existing but previously unrecognized metabolic dysfunction. This limitation precludes causal inference regarding COVID‐19 as the direct trigger of hyperglycemia and reinforces the need for cautious interpretation.

Furthermore, because multiple testing correction was not applied, statistically significant associations should be considered exploratory and hypothesis‐generating rather than definitive genetic discoveries. Replication in larger independent cohorts will be essential to establish reproducibility.

Despite these limitations, this study provides several strengths. To our knowledge, this is among the first studies to investigate associations between innate immune genetic polymorphisms and hyperglycemia in a post‐COVID population from Southeast Asia. The use of a clinically well‐characterized prospective cohort with integrated metabolic, inflammatory, immunological, and host genetic data enabled a multidimensional assessment of immune‐metabolic interactions. These findings contribute to the emerging understanding of host biological factors that may shape heterogeneous metabolic recovery following viral infection.

Several limitations should be acknowledged. First, the absence of pre‐COVID glycemic measurements limits the ability to determine whether hyperglycemia observed during follow‐up represented newly developed metabolic abnormalities or pre‐existing undiagnosed hyperglycemia. Second, the inclusion of participants with established diabetes mellitus and other cardiometabolic comorbidities introduces potential confounding, although multivariable adjustment was performed to reduce this effect. Third, lifestyle‐related factors, including dietary habits, physical activity, and behavioral changes during post‐COVID recovery, were not systematically collected and may have influenced metabolic outcomes [30]. Fourth, because this study was conducted at a single tertiary center in Thailand, generalizability to other ethnic populations and healthcare settings may be limited. Fifth, multiple testing correction was not performed, increasing the possibility of false‐positive associations. Finally, no functional validation experiments were conducted; therefore, the biological consequences of the identified polymorphisms remain speculative.

## 5. Conclusion

In conclusion, this study identified significant associations between polymorphisms in the innate immune genes *NOS2* and *CCL2* and hyperglycemia among individuals recovering from COVID‐19. These findings support the hypothesis that host immune genetic variation may contribute to heterogeneity in metabolic outcomes following viral infection, potentially through inflammatory and immune‐metabolic mechanisms. However, given the observational nature of the study, baseline metabolic confounding, and the exploratory nature of the genetic analyses, these findings should be interpreted cautiously. Independent validation and mechanistic studies are required to clarify the biological relevance and potential clinical implications of these candidate variants.

**Nomenclature**
COVID‐19: coronavirus disease 2019
HWE: Hardy–Weinberg equilibrium
IL: interleukin
MAF: minor allele frequency
PCR: polymerase chain reaction
SNPs: single‐nucleotide polymorphisms

## Author Contributions

Watip Tangjittipokin: conceptualization, funding acquisition, methodology, and writing—review and editing; Tassanee Narkdontri, Suavaluk Songlilitchuwong, and Saranya Innang: data curation; Ganyalak Chaimaha and Nipaporn Teerawattanapong: formal analysis and writing—original draft; Ganyalak Chaimaha, Nipaporn Teerawattanapong, Kaweeraphat Chaithaisong, Tassanee Narkdontri, Suavaluk Songlilitchuwong, Saranya Innang, Sarocha Suthon, Alessia Micieli, and Watip Tangjittipokin: investigation; Sarocha Suthon: methodology and writing—review and editing.

## Funding

This research project was supported by Mahidol University (Fundamental Fund: Fiscal Year 2023, by the National Science Research and Innovation Fund [NSRF]; Grant Number FF‐030/2566). Additional support was provided by the Research Excellence Development (RED) Program, Faculty of Medicine Siriraj Hospital, Mahidol University. The author also acknowledges the support of the Development and Promotion of Science and Technology Talents Project (DPST) scholarship, awarded by the Royal Government of Thailand, as well as the Siriraj Scholarship.

## Ethics Statement

This study was conducted in accordance with the ethical principles of the Declaration of Helsinki and approved by the Siriraj Institutional Review Board (SIRB), Faculty of Medicine Siriraj Hospital, Mahidol University, under Approval Number COA No. Si 225/2022. Written informed consent was obtained from all participants before enrollment. All participant information and genetic data were handled in accordance with institutional confidentiality and data protection regulations.

## Conflicts of Interest

The authors declare no conflicts of interest.

## Data Availability

The datasets generated and/or analyzed during the current study are available from the corresponding author upon reasonable request.
